# Factors associated with cam deformity in Japanese local residents

**DOI:** 10.1038/s41598-024-51876-0

**Published:** 2024-01-18

**Authors:** Koichi Tomomatsu, Takaya Taniguchi, Hiroshi Hashizume, Teiji Harada, Toshiko Iidaka, Yoshiki Asai, Hiroyuki Oka, Shigeyuki Muraki, Toru Akune, Hiroshi Kawaguchi, Kozo Nakamura, Munehito Yoshida, Sakae Tanaka, Noriko Yoshimura, Hiroshi Yamada

**Affiliations:** 1 Naga Municipal Hospital, 1282 Uchida, Kinokawa City, Wakayama Japan; 2 School of Health and Nursing Science, Wakayama Medical University, 590 Mikazura, Wakayama City, Wakayama Japan; 3 Department of Preventive Medicine for Locomotive Organ Disorders, 22nd Century Medical and Research Center, Faculty of Medicine, University of Tokyo, 7‑3‑1 Hongo, Bunkyo‑ku, Tokyo Japan; 4 Department of Orthopaedic Surgery, Wakayama Medical University, 811‑1 Kimiidera, Wakayama City, Wakayama Japan; 5 Department of Medical Research and Management for Musculoskeletal Pain, 22nd Century Medical &Research Center, Faculty of Medicine, University of Tokyo, 7‑3‑1 Hongo, Bunkyo‑ku, Tokyo Japan; 6 National Rehabilitation Center for Persons With Disabilities, 4‑1 Namiki, Tokorozawa City, Saitama Japan; 7 Nadogaya Hospital, 2-1-1Shinkashiwa, Kashiwa, Chiba Japan; 8 Department of Orthopaedic Surgery, Towa Hospital, 4‑7‑10 Towa, Adachi‑ku, Tokyo Japan; 9 Department of Orthopaedic Surgery, Sumiya Orthopedic Hospital, 337 Yoshida, Wakayama City, Wakayama Japan; 10 Department of Orthopaedic Surgery, Faculty of Medicine, University of Tokyo, 7‑3‑1 Hongo, Bunkyo‑ku, Tokyo Japan

**Keywords:** Anatomy, Medical research

## Abstract

Femoroacetabular impingement has increasingly been recognized as a cause of primary hip osteoarthritis; however, its epidemiological indications remain unclear. We aimed to clarify the epidemiological indications and factors associated with cam deformity in a large-scale population-based cohort in Japan. Overall, 1480 participants (2960 hips) (491 men, 989 women; mean age, 65.3 years) analyzed in the third survey of the Research on Osteoarthritis/Osteoporosis Against Disability study were included. The α angle and spinopelvic parameters (lumbar lordosis, sacral slope, pelvic tilt, and pelvic incidence) were radiographically measured. Cam deformity was defined as α angle ≥ 60°. Overall, the cam deformity prevalence was 147/2960 (5.0%). Cam deformity prevalence tended to increase with age; in the univariate analysis, a higher percentage of men was observed in the group with cam deformity than in the group without it. No relationship was observed between cam deformity and hip pain. Factors associated with α angle were examined via multiple regression analysis for each gender; α angle was significantly associated with age and BMI in each gender. The α angle and PT were correlated in women. Thus, α angle and cam deformity prevalence increase with age in Japanese individuals. Accordingly, cam deformity can be considered a developmental disease.

## Introduction

Ganz et al.^1^ defined the mechanisms of cartilage damage associated with femoroacetabular impingement (FAI) as well as its clinical and radiographic features. FAI is caused by three anatomic abnormalities. Cam-type FAI is characterized by the asphericity of the femoral head and lack of femoral offset at the femoral head-neck junction^1^. Pincer-type FAI is associated with acetabular over-coverage. Mixed-type FAI includes the characteristics of cam- and pincer-type FAI. In recent years, cam-type FAI has been proposed as a biomechanical risk factor for hip osteoarthritis (OA)^2,3^ and total hip arthroplasty (THA)^4^; however, the epidemiological factors associated with cam deformity remain unclear in Japanese people. In a past study, we reported the prevalence of pistol grip deformity (PGD) and related factors in the Japanese population^5^. This population-based cohort study included 1575 Japanese local residents. It showed that the prevalence of PGD in Japanese people was 4.9% (10.6% in men and 2.1% in women), with the prevalence increasing with age. Analysis of factors associated with the presence of PGD revealed significant differences in gender, age, and BMI. It is possible that spinopelvic parameters affected acquired factors in the hip with age but had no significant effects on PGD in the previous study. However, in the multivariate analysis the *p*-value of PGD and pelvic tilt (PT) was 0.0746, suggesting that PT may influence PGD. Since the presence or absence of PGD is qualitative, the quantitative evaluation of cam deformity is difficult. Therefore, in this study we used the α angle, which can quantitatively measure cam deformity, to analyze the prevalence of cam deformity in the Japanese population and its related factors, including spinopelvic parameters.

## Results

### Prevalence of cam deformity

Table 1 shows the prevalence of cam deformities in the overall study population and the age subgroups. In the overall study population, the prevalence of cam deformity was 147/2960 (5.0%). The prevalence of cam deformities significantly increased with age (Table 1).

**Table 1:** Prevalence of cam deformity in the five age groups. The prevalence of cam deformity tended to increase with age (Cochran–Armitage test; *p* < 0.0001).

Age group (years)	Cam deformity (+) (hips)
≤ 49	9/352 (2.6%)
50–59	8/528 (1.5%)
60–69	30/842 (3.6%)
70–79	55/826 (6.7%)
≥ 80	45/412 (10.9%)
Total	147/2960 (5.0%)

### Comparison of the demographics between groups with and without cam deformity

The cam deformity-positive group comprised a significantly higher proportion of men and older persons than the cam deformity-negative group (Table 2).

**Table 2:** Comparison of the demographics and spinopelvic parameters between the groups with and without cam deformity. Results are presented as mean ± standard deviation (95% confidence intervals).

	Cam deformity (+)	Cam deformity (–)	*p*-value
Number of participants (hips)	108 (147)	1372 (2813)	
Demographics			
Age	72.5 ± 12.7 (70.5–74.6)	65.0 ± 12.9 (64.5–65.4)	< 0.0001^+^
Sex (men vs. women) (hips)	105 vs. 42	877 vs. 1936	< 0.0001*
Hip pain	1/147	56/2813	0.2597
BMI	23.5 ± 3.4 (23.0–24.0)	23.0 ± 3.5 (22.8–23.1)	0.0567
Spinopelvic parameters			
LL	42.0 ± 17.3 (39.1–44.8)	45.6 ± 13.4 (45.1–46.1)	0.0015^+^
SS	30.0 ± 10.6 (28.2–31.7)	31.8 ± 9.0 (31.5–32.2)	0.0151^+^
PT	19.8 ± 10.0 (18.2–21.4)	18.1 ± 9.2 (17.8–18.4)	0.0290^+^
PI	49.8 ± 11.3 (47.9–51.6)	50.0 ± 10.6 (49.6–50.3)	0.845

BMI: body mass index, LL: lumbar lordosis, SS: sacral slope, PT: pelvic tilt, PI: pelvic incidence.

*Significant group difference by the chi-square test. ^+^Significant group difference by the non-paired t-test.

### Association of cam deformity with hip pain

Among the 147 cases of cam deformity-positive hips, hip pain on the affected side was reported in only one case. No significant association was observed between cam deformity and hip pain (Table 2).

### Association of cam deformity with spinopelvic parameters

Lumbar lordosis (LL) and sacral slope (SS) were significantly lower (*p* = 0.0015 and *p* = 0.0151, respectively) and PT was significantly higher in the group with cam deformity (*p* = 0.0290) (Table 2).

### Factors associated with the α angle

**Figure 1:**
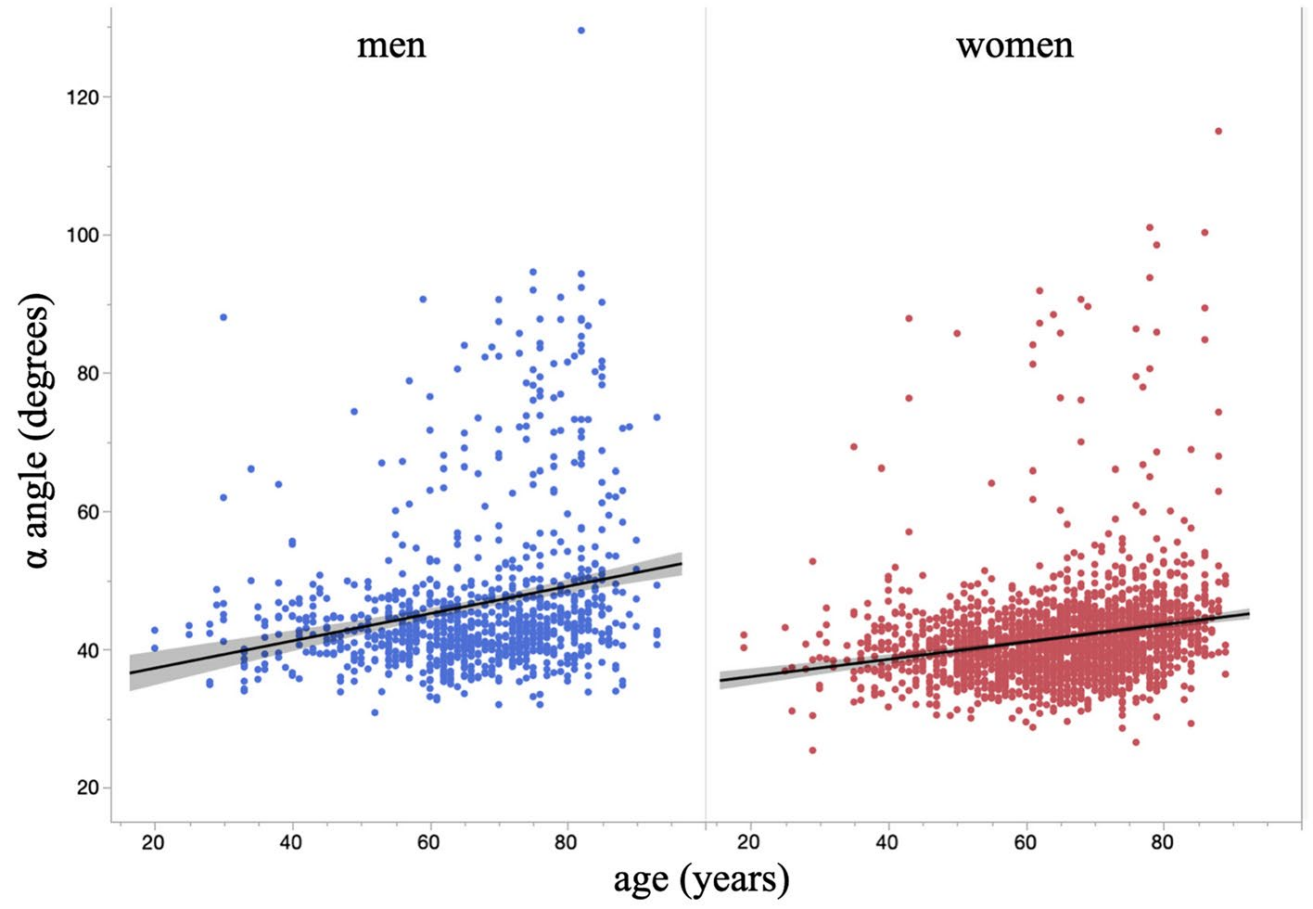
Scatterplot of the α angle and age.

Multiple regression analysis with the α angle as the objective variable and age, body mass index (BMI), LL, PT, and PI as explanatory variables was conducted to investigate the factors associated with the α angle for each gender (Table 3). The α angle was significantly associated with age (Table 3). Further, the α angle increased with age (Fig. 1). There was a correlation between α angle and BMI in each gender and between α angle and PT in women (Table 3).

**Table 3:** Factors associated with the α angle.

	Standardized β	*p*-value	VIF
Men			
Age	0.25	< 0.0001*	1.19
BMI	0.09	0.0058*	1.06
LL	− 0.10	0.0751	3.35
PT	− 0.05	0.4122	3.99
PI	0.08	0.1962	4.33
Women			
Age	0.15	< 0.0001*	1.23
BMI	0.07	0.0009*	1.02
LL	0.02	0.548	3.24
PT	0.16	0.0004*	4.56
PI	− 0.06	0.1698	4.33

VIF: variance inflation factor, BMI: body mass index, LL: lumbar lordosis, PT: pelvic tilt, PI: pelvic incidence.

*Significant association with the α angle by multiple regression analysis.

## Discussion

Several measures have been proposed to quantify cam deformities^2^. The α angle is the most commonly used method and was first described based on axial-plane magnetic resonance images^6^. In several studies, anteroposterior radiography of both hips was used to measure the α angle^2,3^. Agricola et al.^3^ measured the α angle in anteroposterior radiographs of both hips and reported that the greater the α angle, the higher the risk of hip OA. Moreover, these studies agreed that the radiographic presence of cam deformity was defined by an α angle greater than 60°^2,3^.

In the present study, the overall prevalence of cam deformities was 5.0%. In another population-based study conducted in Japan by Hasegawa et al., the overall prevalence of cam deformity was 4.2%^7^. In Europe and the United States, the prevalence of cam deformities can be radiographically found to be 15%–25% in men and 0%–15% in women^8,9^, and it tends to be higher than that in Japan. Similar to our results, several previous studies have reported that cam deformities are more common in men than in women^7,8^. According to reports from Western countries, men tend to have a smaller femoral head-neck offset and a smaller combined version, which can lead to FAI^10^. Similar biomechanics may also apply to the Japanese population.

In this study, the presence of cam deformity was not correlated with hip pain. Hasegawa et al.^7^ also reported no correlation between FAI and hip pain in the Japanese population. Radiographic cam deformities do not always cause impingement^3^. Gosvig et al.^8^ reported that hip pain occurred in 16% of the patients with cam deformities. Thus, there may be fewer symptomatic patients with cam deformities in Japan than in Europe or the United States. Nakamura et al.^11^ reported that the acetabular roofs of Japanese individuals are shallower than those of Europeans and Americans. Takeyama et al.^12^ reported that the anteroposterior diameter of the femoral neck was smaller in Japanese individuals than in Europeans and Americans. Thus, FAI is less likely to occur in Japanese patients because of differences in hip anatomy. Although the number of surgeries for cam-type FAI has increased in recent years, it is important to note that cam deformity is not associated with pain in Japanese patients.

The most notable result of this study was that the α angle increased with age. The α angle was significantly associated with age. Allen et al.^13^ investigated 113 patients with cam deformities and suggested that the deformities may be congenital, whereas Agricola et al.^1,2^ suggested that cam deformities are related to high-level sports activities in adolescents. However, the pathology of cam deformities has not been elucidated. We reported the prevalence of PGD and related factors among the same participants^5^. However, the prevalence of PGD is low in Japanese individuals, and the quantitative evaluation of PGD is difficult. The presence or absence of PGD did not allow for the examination of factors associated with cam-type FAI, and we could not evaluate the PGD-negative group in our previous study^5^. Therefore, to quantitatively evaluate the asphericity of the femoral head, we measured the α angle in anteroposterior radiographs of both hips, and the radiographic presence of a cam deformity was defined as an α angle greater than 60°. The prevalence of cam deformity increased with age, and, surprisingly, it was found that the α angle increased with age in all participants, including those who did not meet the criteria for cam deformity. Further, Resnick et al.^14^ suggested that deformation of the femoral head could be caused by remodeling. It is expected that the α angle may increase because of the load given that the α angle has a slight positive correlation with BMI.

Weinberg et al.^15^ found that FAI patients have a lower pelvic incidence than healthy controls. Husson et al.^16^ reported that FAI patients and healthy controls exhibited similar spinopelvic parameters. The spinopelvic parameters in FAI patients have not been fully elucidated. Considering gender differences, we performed multivariate analysis separately for men and women in this study. Unlike the previous PGD study, this study showed an association between the α angle and PT in women significantly. The α angle and cam deformity showed different correlations with spinopelvic parameters in men and women. To avoid impingement, PT may increase with the development of cam deformity in women.

This study has some limitations. First, causal relationships between the evaluated variables could not be determined because of the cross-sectional study design. Thus, a follow-up longitudinal study is required to confirm the findings of this study. Second, cam deformity was assessed only by measuring the α angle in anteroposterior radiographs of both hips under weight-bearing conditions. Cam deformity is a three-dimensional abnormality centered anterosuperiorly; therefore, the α angle can depend on the view used to measure it^2^. Finally, selection bias may exist. Because participants with a history of hip surgery were excluded from this study, patients with severe OA or other hip lesions might not have been included. Moreover, regional selection bias should be considered because the participants (voluntary participants) were recruited from only two regions. Notably, Yoshimura et al. reported that participants in the ROAD study were representative of the Japanese population^17^. However, these findings may not be generalizable to other populations. Accordingly, applying the results of this study to different races or countries with people of different lifestyles would require careful judgment.

In conclusion, cam deformities are prevalent in the general population in Japan; however, they do not correlate with hip pain. Cam deformities and spinopelvic parameters may be differentially correlated between men and women. The prevalence of cam deformity and α angle increases with age, suggesting that cam deformity may be a developmental disease in Japanese local residents.

## Methods

### Participants

The Research on Osteoarthritis/Osteoporosis Against Disability (ROAD) study is a large-scale population-based cohort study of bone and joint diseases conducted in three communities in Japan: an urban region in Itabashi, Tokyo, with a population of 529,400/32 km^2^; a mountainous region in Hidakagawa, Wakayama, with a population of 11,300/330 km^2^; and a coastal region in Taiji, Wakayama, with a population of 3500/6 km^2^. A detailed profile of the ROAD study has been previously reported^17–20^. This study included participants from the third survey of the ROAD study involving mountainous and coastal regions.

The third survey of the ROAD study was conducted between October 2012 and December 2013. In addition to the previous participants, inhabitants of the mountainous and coastal areas in the Wakayama Prefecture who were willing to participate in the study were also included. Of the 1575 individuals (513 men and 1062 women) who initially participated in the survey, 22 with a history of hip surgery because of OA or proximal femoral fracture were excluded because of changes in hip morphology. Further, 59 individuals did not undergo hip or whole-spine radiography. In addition, eight individuals had indistinct radiographs or hip OA (Kellgren–Lawrence grade ≥ 3), and six had incomplete data on hip pain. The remaining 1480 participants (2960 hips) (491 men and 989 women; mean age, 65.3 years) were included in this study. The inclusion and exclusion processes detailed above were based on a previous study^5^. Participant characteristics are listed in Table 4.

**Table 4:** Demographics of the participants. Values are presented as mean ± standard deviation.

	Total	Men	Women
Number of participants	1,480	491	989
Age (years)	65.3 ± 13.0	66.2 ± 13.8	64.9 ± 12.5
Height (cm)	156 ± 9.1	165 ± 7.2	152 ± 6.6
Weight (kg)	56.4 ± 11.3	64.6 ± 13.0	62.4 ± 8.7
BMI (kg/m^2^)	23.0 ± 3.5	23.7 ± 3.5	22.7 ± 3.5

BMI, body mass index.

The participants documented their family medical history, personal medical history, and history of hip surgery through an interviewer-administered questionnaire. Physical parameters, including height and weight, were recorded, and BMI was calculated. Furthermore, all participants were assessed by well-experienced orthopedists for the presence of hip pain, which was defined as the presence of any hip pain in most parts of the past month, in addition to current pain.

### Radiographic evaluation

**Figure 2:**
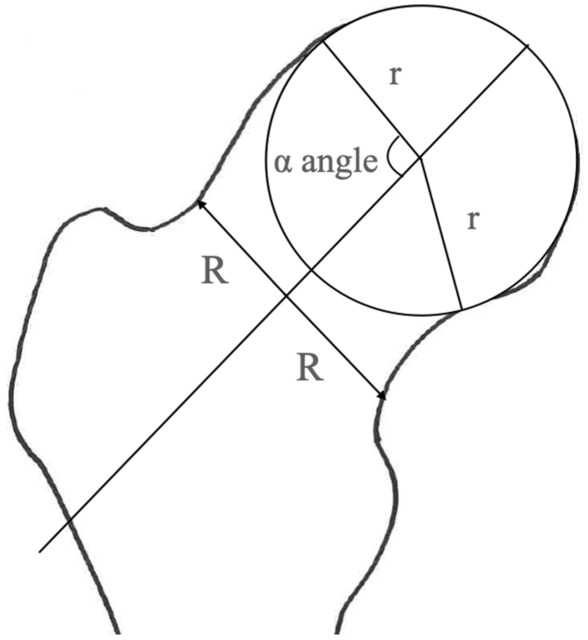
Measurement of the α angle ^2,3^.

The methods described in this section were according to those in a previous paper^21^. Briefly, all the participants underwent radiographic examinations. Both hips underwent anteroposterior radiography under weight-bearing conditions. A well-experienced orthopedist (K.T.) who was blinded to the clinical status of the participants assessed the hip radiographs, and the α angle was determined. The α angle was measured by first drawing the best-fitting circle around the femoral head, then a line through the center of the neck and the center of the head. From the center of the femoral head, a second line was drawn to the point where the superior surface of the head-neck junction first departed from the circle. The angle between these two lines was then measured as the α angle (Fig. 2)^2,3^. We defined the radiographic presence of cam deformity based on an α angle greater than 60°. To evaluate the intra-observer reliability of the α angle, 50 randomly selected hip radiographs were analyzed by the same observer more than 1 month after the first reading. The intra-observer reliability of the α angle was 0.85. Moreover, standing lateral radiographs of the whole spine and pelvis were obtained to determine the spinopelvic alignment parameters. Each radiograph was aligned such that the edge of the film was used as a reference for vertical alignment.

**Figure 3:**
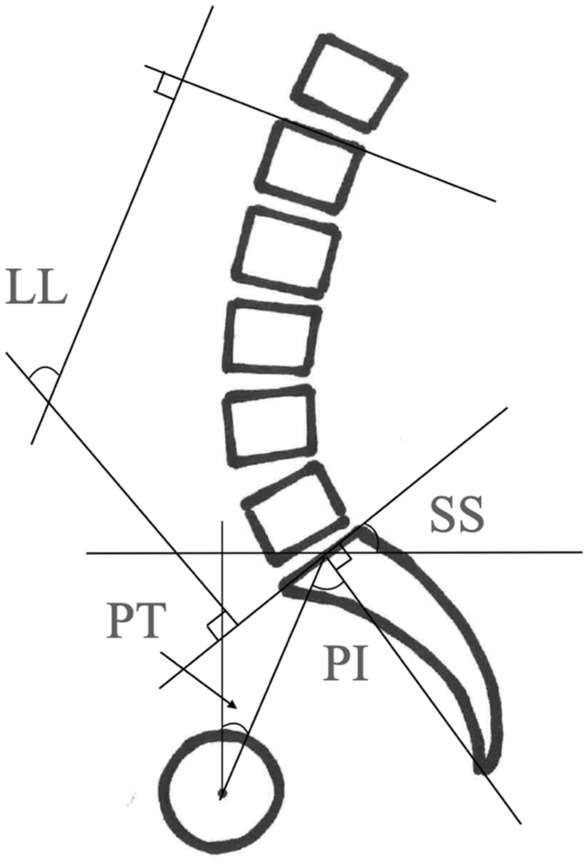
Schema of the measuring method for each spinopelvic parameter. LL, lumbar lordosis; SS, sacral slope; PI, pelvic incidence; PT, pelvic tilt.

On standing lateral radiographs, the following parameters were measured: LL (the Cobb angle from the upper endplate of L1 to the lower endplate of S1), SS (the angle between the tangent line to the superior endplate of S1 and the horizontal plane), PT (the angle between the line connecting the midpoint of the sacral plate to the axes of the femoral heads and the vertical axis), and PI (the angle between the line perpendicular to the sacral plate at its midpoint and the line connecting this point to the axes of the femoral heads) (Fig. 3).

To evaluate the intra-observer reliability of the spinopelvic parameters, 50 randomly selected whole-spine radiographs were assessed by the same observer more than 1 month after the first measurement, whereas another 50 radiographs were analyzed by two experienced orthopedists (T.H. and Y.A.) to evaluate inter-observer reliability. The intra-observer and inter-observer reliabilities based on intraclass correlation coefficients were adequate for assessment (LL, 0.96 and 0.94; SS, 0.91 and 0.93; PT, 0.99 and 0.97; PI, 0.93 and 0.91, respectively)^21,22^.

### Statistical analysis

Statistical analyses were performed using JMP software (version 16; SAS Institute Inc., Cary, NC, USA). Quantitative values are expressed as mean ± standard deviation and 95% confidence intervals. Participants were classified into five age groups based on their birth year: (1) < 50 years, (2) 50–59 years, (3) 60–69 years, (4) 70–79 years, and (5) ≥ 80 years. The Cochran–Armitage test was conducted to determine trends in cam deformity prevalence among the five age groups. The chi-square test was performed to compare the presence of cam deformity between men and women. Differences in age, BMI, and sagittal spinopelvic parameters (LL, SS, PT, and PI) between groups with and without cam deformity were evaluated using a non-paired t-test. Multiple regression analysis was conducted to examine the association of age, BMI, and sagittal spinopelvic parameters (LL, PT, and PI) with the α angle for each gender. A scatterplot of the α angle and age was created.

### Ethics declarations

All participants provided written informed consent for their participation and the publication of the study via print and electronic forms. This study, establishment of the cohort and study design, was approved by the Research Ethics Review Committee of the University of Tokyo (no. 1326). The procedures followed were in accordance with the ethical standards of the responsible committee on human experimentation (institutional and national) and the Declaration of Helsinki of 1975 revised in 2000.

## Data Availability

All data generated or analyzed in this study are available from the corresponding author upon reasonable request.
